# AI-based patient monitoring for fall prevention in stroke patients: a pilot study at a Malaysian acute stroke unit

**DOI:** 10.1186/s12984-025-01706-9

**Published:** 2025-10-21

**Authors:** Monica Danial, Chee Toong Chow, Meng Hui Lim, Noor Azleen Ayop, Irene Looi, Alan Swee Hock Ch’ng

**Affiliations:** 1 Clinical Research Centre (CRC) Hospital Seberang Jaya, Institute for Clinical Research, Ministry of Health Malaysia (MOH), Seberang Jaya, Penang, Malaysia; 2 Department of Emergency and Trauma Hospital Seberang Jaya, Ministry of Health Malaysia (MOH), Seberang Jaya, Penang, Malaysia; 3 SmartPeep Pte Ltd, Singapore, Singapore; 4 Medical Department, Hospital Seberang Jaya, Ministry of Health Malaysia (MOH), Seberang Jaya, Penang, Malaysia

**Keywords:** SMART AI patient sitter system, AI-based monitoring, Fall, Hospitalized patients, Stroke

## Abstract

**Background:**

Falls are an important patient safety concern and stroke patient are at high risk. Artificial intelligence (AI) could be leveraged to reduce patient falls in the hospital but there is scarcity of data. Therefore, the aim of this study is to evaluate the effectiveness of the SMART AI Patient Sitter system—an AI-powered motion-sensing and alert system designed for fall detection and prevention in a real-world hospital setting.

**Methods:**

Conducted from January to December 2024 at the Acute Stroke Unit of Hospital Seberang Jaya (ASUHSJ), the study involved 30 stroke patients who consented to AI monitoring. The SMART AI patient sitter system comprised an optical sensor, alert panel, and control panel monitored by AI, which detected patient movement and triggered alerts to the observation counter. Blurred, non-identifiable images maintained patient privacy, and investigators were identified through uniform recognition. Data on mobility and fall events were recorded continuously.

**Results:**

The integration of this system led to an 83.33% reduction in fall incidents and the generation of 1,439 alerts with a 95.34% accuracy rate. Enrolled patients had a mean age of 61 years(SD ± 12.8) years; 63.3% were male; 56.7% were of Malay ethnicity and 83.3% were classified as high fall risk. The median duration of monitoring was 3 days (IQR: 1.0–6.0), with a median of 19 bed exits(IQR: 1.0–85.0) bed exits. The first bed exit attempt occurred at a median of 150 minutes (IQR: 20.0–2103.0) minutes post-admission. Response time to movement alerts was prompt, with a median of 21  seconds (IQR: 4.0–75.0). Only one fall (3.3%) was recorded during the study. The incident involved a moderate-risk patient who attempted to stand abruptly. Staff responded within 29 seconds, and the patient recovered without severe injury.

**Conclusion:**

These findings suggest the system’s potential in early detection and timely intervention. Study data demonstrated wide variability in patient mobility patterns, highlighting the need for individualized monitoring. The SMART AI patient sitter system’s ability to deliver real-time alerts, ensure patient privacy, and reduce fall incidence demonstrates its value in improving stroke patient safety. Overall, this study supports the integration of AI-based monitoring tools in clinical settings to enhance patient care and reduce preventable incidents like falls.

## Background

Falls are the most common safety incident among hospitalized patients, with fall rates from 2.9 to 13 per 1,000 patient days [1, 2]. Falls can lead to serious consequences, including fractures, head injuries, and prolonged hospital stays, complicating rehabilitation efforts [3]. Fall incidence among stroke patients is a significant concern due to their impaired mobility, muscle weakness, and cognitive deficits [4]. Studies indicate that approximately 30% of stroke survivors experience at least one fall annually, with 15% falling twice or more [5]. This heightened risk is particularly pronounced during the early stages of recovery as patients strive to regain independence. Factors such as balance impairment, poor coordination, and reduced sensation further contribute to this vulnerability [6]. Preventive strategies like continuous monitoring, assistive technologies, and early mobility training are crucial in reducing fall incidence among stroke patients. Effective fall prevention not only enhances patient safety but also improves recovery outcomes and overall quality of life.

Falls among stroke survivors in Malaysia are a significant concern, though specific national data are limited. Research indicates that approximately 37% of stroke survivors experience at least one fall within six months post-stroke. Additionally, a study conducted in a Malaysian hospital reported an incidence of falls at 1.0 per 1,000 patient-days among hospitalized older patients, highlighting the vulnerability of this population [7]. Our hospital data shows an increase in total falls in 2024, with approximately 49 incidents reported, compared to 37 incidents in 2023 across the entire hospital. There was also an increase in total stroke admissions in 2024, with approximately 888 cases compared to 855 cases in 2023.

Various strategies have been implemented in hospital settings to mitigate the risk of patient falls. Physical restraints, such as bed rails, belts, and chairs with locking trays, have traditionally been used to prevent unassisted movement [8]. However, these measures are increasingly discouraged due to ethical concerns and the risk of injury, agitation, or functional decline [9]. Medical restraints, typically involving the use of sedatives or antipsychotic medications, are also controversial and may impair cognitive and physical function, thus potentially increasing fall risk [10]. As a safer and more ethical alternative, virtual restraints including sensor-based alert systems and AI-powered video monitoring are gaining traction. These systems provide real-time movement detection and alerts without limiting the patient’s physical autonomy [11]. For example, Mao et al., 2023 developed *eNightTrack*, a restraint-free, depth-camera-based surveillance and alarm system that utilizes deep learning to track patient movement and prevent falls in hospitals [12]. Compared to traditional methods, virtual restraints offer a proactive and less intrusive way to monitor high-risk patients, enabling timely staff interventions while preserving patient dignity [13].

Recent advancements in state-of-the-art (SOTA) fall monitoring technologies have expanded beyond video-only approaches to include a broad range of sensor modalities [14]. Wearable sensors, such as inertial measurement units (IMUs) are popular due to their portability and motion-tracking capabilities however, they require patient compliance, which is particularly challenging among stroke patients with cognitive or mobility impairments [15]. Electromyography (EMG) and electrocardiography (ECG) sensors have been studied for detecting physiological changes during pre-fall phases, but they are often sensitive to noise and require direct skin contact or placement in specific locations, limiting their clinical practicality [16]. Micro-electromechanical systems (MEMS) technology has improved miniaturization of these sensors, but the need for charging and maintenance adds operational complexity in hospitals [14]. Meanwhile, remote sensing technologies such as radar, thermal imaging, and depth sensors allow for contactless monitoring, yet they frequently suffer from high false alarm rates, privacy concerns, and hardware costs that hinder large-scale adoption [17].

Although various fall risk factors have been identified, limited research has focused on automatic sensing systems within hospital rooms for detecting falls, assessing fall risks, and analyzing pre-fall events [17]. An emerging technological approach is the SMART AI Patient Sitter system, an AI-enabled solution that utilizes ceiling-mounted optical sensors to monitor patient activity. Video data is transmitted to a centralized server, where software algorithms perform real-time analysis to detect potentially high-risk events, such as unassisted bed exits by patients at elevated fall risk or in-room falls. The system is designed to concurrently monitor multiple beds across different cubicles or wards.

The aim of this study is to evaluate the implementation of the SMART AI Patient Sitter system, for fall detection and prevention in hospitalized stroke patients. By deploying this system in real hospital settings, the research aims to assess its effectiveness in providing a proactive and non-intrusive solution for fall prevention and detection. The findings from this study could offer valuable insights into enhancing patient safety through advanced sensor technology.

## Methods

### Study setting

This study was conducted at the Acute Stroke Unit of Hospital Seberang Jaya (ASUHSJ), a specialized six-bed unit dedicated to the care of acute stroke patients. ASUHSJ was chosen due to its high patient activity levels and the increased risk of falls among its patients.

### SMART AI patient sitter system

**Fig. 1:**
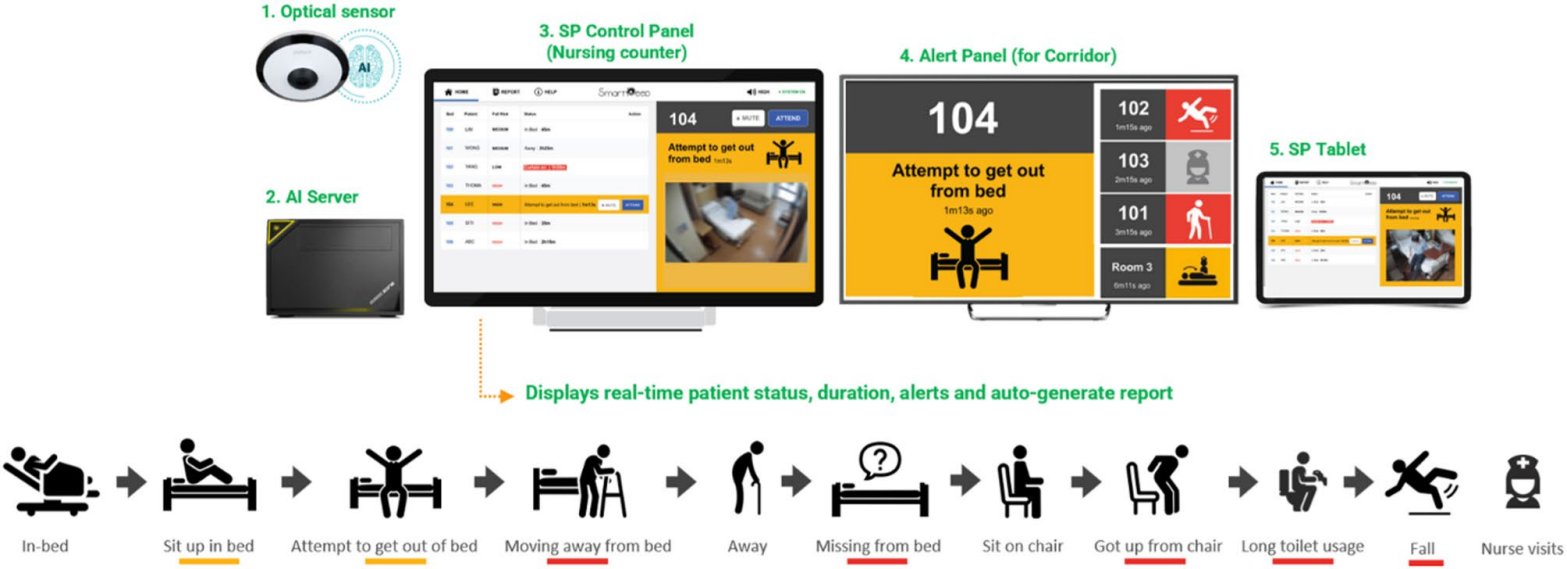
The SMART AI patient sitter system

The SMART AI Patient Sitter system, an artificial intelligence-powered solution designed for fall detection and prevention was developed by SmartPeep, a health technology company based in Singapore. The system enables continuous, non-contact monitoring of patients within hospital settings. The system comprises four key components: optical sensors, a centralized AI engine, control and alert panels, and a secure integration interface to support workflows (Fig. 1).

The optical sensor, typically mounted on the ceiling or high on a wall, captures wide-angle visual data of the patient’s bed area. It supports standard monitoring formats, including fisheye and thermal imaging, and transmits real-time video streams to a local AI server. The sensor is positioned to detect postural changes and movement patterns, such as sitting up or attempting to leave the bed, while limiting the capture of facial details to support patient privacy.

The AI engine, hosted on either a local or centralized server, runs advanced computer vision algorithms to analyze motion in real time. It is capable of detecting high-risk behaviors such as unassisted bed-exits, extended time spent near the bed edge, and potential falls. By learning spatial and temporal movement cues, the system distinguishes between routine activity and actual risk, thereby reducing false alarms. When an anomaly is identified, the AI generates a color-coded alert (for example: red for urgent or amber for warning) to facilitate rapid and prioritized staff response.

The control panel, located at the observation counters, provides a dashboard view of all monitored beds. Blurred preview images are displayed, deliberately anonymized to protect patient identity while preserving key visual indicators such as posture and movement. The interface allows staff to monitor alert history, and to activate or deactivate patient monitoring based on consent and clinical needs.

**Fig. 2:**
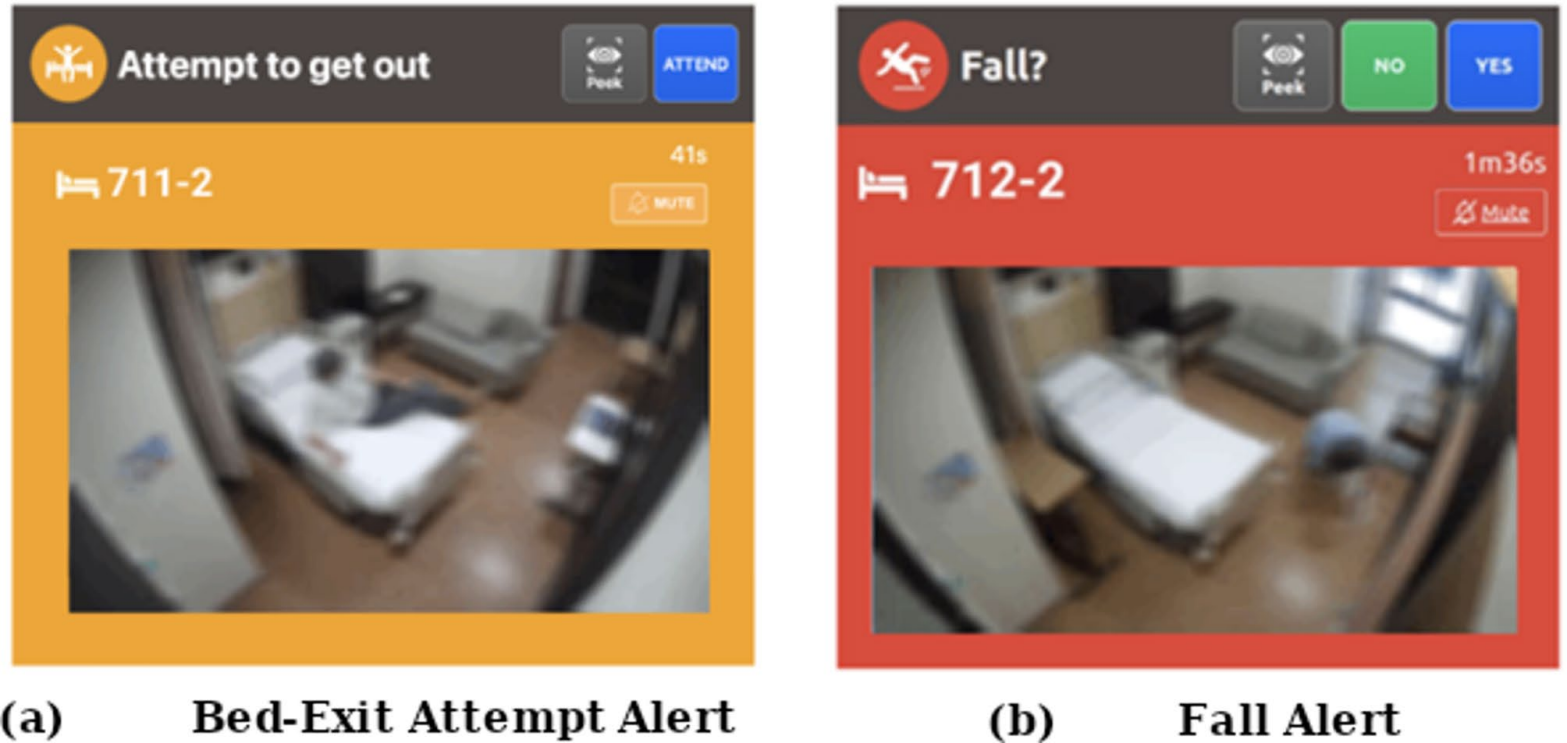
Blurred visual output displayed on the control panel at the observation counter when a patient (**a**) attempts to leave the bed, and (**b**) falls in the room

Figure 2 show examples of the image outputs on the control panels for a bed-exit attempt and a fall. The design allows for the capture of essential patient activity data while reducing the visibility of identifiable facial features, supporting both patient safety and data confidentiality.

To further support staff responsiveness, an alert panel is installed outside patient rooms. Mounted on the corridor ceiling, this panel uses visual indicators such as icons and colored lights to signal the priority of alerts and guide staff to the appropriate room, even when they are away from the central monitoring station. This facilitates faster incident localization and decision-making during high-activity periods.

The system also incorporates staff presence recognition by detecting uniformed staffs to automatically acknowledge and dismiss alerts upon arrival, eliminating the need for manual confirmation. This feature enables automated logging of response times, which can support performance evaluation and workflow optimization.

In addition, the system’s secure integration interface ensures compatibility with hospital infrastructure and clinical workflows. This includes adherence to data privacy protocols, compliance with healthcare information technology (IT) standards, and interoperability with existing alert systems and electronic health records.

Collectively, these components contribute to a comprehensive, privacy-conscious AI platform that enhances fall prevention, streamlines response efforts, and enables monitoring of care processes particularly in high-risk settings such as acute stroke or geriatric wards.

### Fall risk-based sensitivity configuration

**Fig. 3:**
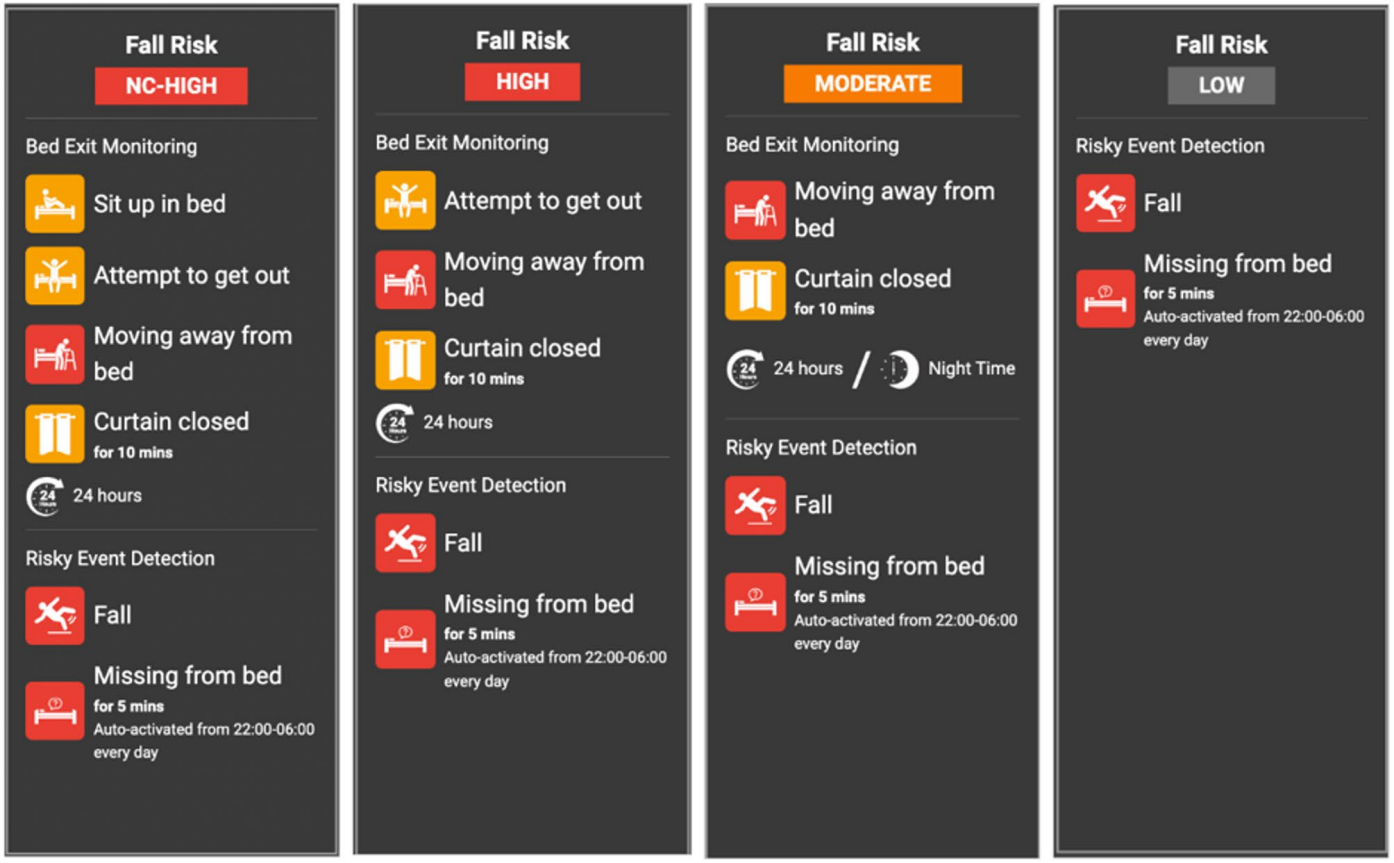
Risk-based alert sensitivity settings for patient monitoring

Figure 3 illustrates how the SMART AI Patient Sitter system adjusts its monitoring sensitivity according to each patient’s assessed fall risk level. Patient monitoring is categorized into four category NC-high (non-compliant high), high, moderate, and low, each with its corresponding alert thresholds and event triggers.

Staff register each patient in the system based on their fall risk assessment, allowing for individualized configuration of monitoring parameters. For instance, high-risk patients are monitored for early indicators such as sitting up, attempting to exit the bed, or moving away from the bedside, while low-risk patients are primarily monitored for critical incidents, such as actual falls or prolonged absence from bed during nighttime hours.

This risk-stratified alert framework supports more efficient triaging of responses, enabling staff to prioritize attention for individuals at highest risk while reducing unnecessary alerts and mitigating alarm fatigue.

### Deep learning framework for patient monitoring

The SMART AI Patient Sitter system employs a vision-based deep learning framework designed for real-time detection of bed exits, falls, and staff presence, utilizing standard optical sensors within hospital environments. For detecting bed exits and falls, the system analyzes video frames captured by ceiling or wall-mounted sensors focused on the patient’s bedside area. Instead of relying on pose estimation, a ResNet-based convolutional neural network (CNN) is used to extract spatial features from each frame. This model identifies behavioral patterns such as lying down, sitting at the edge of the bed, or moving away from the bed and classifies the corresponding frame accordingly.

To detect the presence of staff at the bedside, the system incorporates a You Only Look Once (YOLO)-based object detection model trained to recognize the staff through features such as uniforms or contextual visual indicators. This functionality enables automatic cancellation of alerts when staff are present and supports passive logging of response times, thus eliminating the need for manual input.

Notably, the system avoids the use of resource-intensive architectures such as recurrent neural networks (RNNs) or long short-term memory (LSTM) models for temporal analysis. These were excluded due to their high computational and memory demands, which could increase operational costs and hinder scalability. Instead, a custom event logic engine integrates the CNN’s frame-level outputs with time-based heuristics to detect high-risk patterns, such as approaching the bed edge or initiating bed departure. This combination of deep CNN-based spatial analysis and lightweight temporal logic enables efficient, accurate, and scalable continuous monitoring and supporting timely alerts while maintaining low hardware requirements.

### Study design and data collection

The study was conducted from January to December 2024, with continuous data collection on falls identified by the SMART AI Patient Sitter system. The system displayed real-time patient status and fall risk, functioning according to its intended use. Its alert panel provided various notifications regarding the patient’s condition, with alert colors changing based on priority levels. When an incident or anomaly was detected, the AI server promptly alerted the ASUHSJ observation counter for immediate response.

The investigators involved in the study were clinical staff who were part of the hospital’s regular healthcare team. They continued performing their routine clinical duties throughout the study and were not employed specifically for research purposes. Their responsibilities included documenting fall-related incidents and contributing relevant clinical observations during the course of patient care.

During the signing of consent, patients were informed by the investigators that they would be monitored by AI and that a blurred video would appear at the observation counter when an alert was triggered. Once an alert was activated, the system began counting and awaited the investigator’s arrival in the room to provide assistance. When the investigator arrived and was detected by the system through uniform recognition, the system automatically dismissed the alert and recorded the response time. The interaction was solely between the system and the investigators, without direct engagement with the patients. The system at the ASUHSJ observation counter functioned only as a tool to notify investigators, ensuring timely intervention to prevent falls. The device was activated by an investigator at the counter only after the patient had given consent, initiating fall monitoring for that patient.

Several user training sessions were conducted by the sponsor to familiarize investigators with the system. Investigators were informed that their presence at the patient’s bedside would be tracked through uniform recognition, without the use of personal identifiers or clear facial images. When a subject was discharged from the ward, the investigators was required to check the patient out of the system at the observation counter, with all actions being logged. If a new patient occupied the same bed and required AI monitoring, the investigator had to perform a new check-in with the updated patient details.

AI monitoring was deactivated when the investigator turned off monitoring through the control panel at the investigator counter. After deactivation, all monitoring, alert triggering, and recording ceased. Patients who did not provide consent were not checked into the system, ensuring that no monitoring or alerts were activated for those patients.

### Inclusion criteria and exclusion criteria

Stroke patients aged 18 and above who were admitted to ASUHSJ and agreed to participate in the study, either by themselves or through their legally authorized representative (LAR), were included. Patients or LARs who did not provide consent were excluded from the study. AI monitoring was not conducted on patients who were unwilling to participate. In cases where patients were unable to provide consent themselves, their LARs were approached for consent. However, if a patient later regained consciousness and chose not to participate, the investigator can check the patient out of the system.

### Sample size

All stroke patients admitted to ASUHSJ from January till December 2024.

### Statistical analysis

Continuous variables were summarized using descriptive statistics, including mean, median, standard deviation, minimum, and maximum values. All statistical analyses were calculated using SPSS (version 23; SPSS, Inc., Chicago, IL) statistical software.

## Results

In 2024, a total of 737 patients were admitted to ASUHSJ, of whom only 30 provided consent to participate in this study. The consent-taking process proved to be challenging and time-consuming, primarily due to the high patient turnover at ASUHSJ, which made it difficult to consistently engage eligible participants. Fall risk was evaluated upon admission using the Morse Fall Scale, a widely adopted clinical assessment tool that considers multiple risk factors, including previous falls, presence of secondary diagnoses, use of ambulatory aids, intravenous therapy, gait characteristics, and mental status.

**Table 1:** Demographic profile of SMART AI sitter study participants admitted to ASUHSJ (*n* = 30)

Characteristics	No. (%) of participants	
	*N*	%
**Demographics**		
**Age (years)**, mean (SD)	61	12.8
**Gender**		
Male	19	63.3
Female	11	36.7
**Ethnicity**		
Malay	17	56.7
Chinese	9	30
Indian	4	13.3
**Fall risk at admission**		
Moderate	5	16.7
High	25	83.3
**Fall recorded**		
No	29	96.7
Yes	1	3.3
**Total monitored days**, median (IQR)	3	1.0–6.0
**Total bed exits (number)**, median (IQR)	19	1.0–85.0
**First attempt to get out of bed post admission (minutes)**, median (IQR)	150	20.0-2103.00
**Total attempts to get out of bed (number)**, median (IQR)	117	20.0-2103.00
**Duration prior receiving assistance (seconds)**, median (IQR)	21	4.0–75.0

The Table 1 presents the demographic and clinical characteristics of patients enrolled in the SMART AI Patient Sitter system at the ASUHSJ in 2024. A total of 30 consented stroke patients were enrolled in this study. The patients had a mean age of 61 years with a standard deviation of (± 12.8 years). In terms of gender distribution, 63.3% were male (19 patients), while 36.7% were female (11 patients). Ethnic representation included 56.7% Malay (17 patients), 30% Chinese (9 patients), and 13.3% Indian (4 patients). Based on Morse Fall Scale assessment 83.3% of patients were categorized as high fall risk, while 16.7% were classified as moderate risk. The monitoring data showed that patients were observed for a median duration of 3 days (IQR:1.0 to 6.0). The median number of total bed exits was 19 (IQR: 1.0–85.0).

**Fig. 4:**
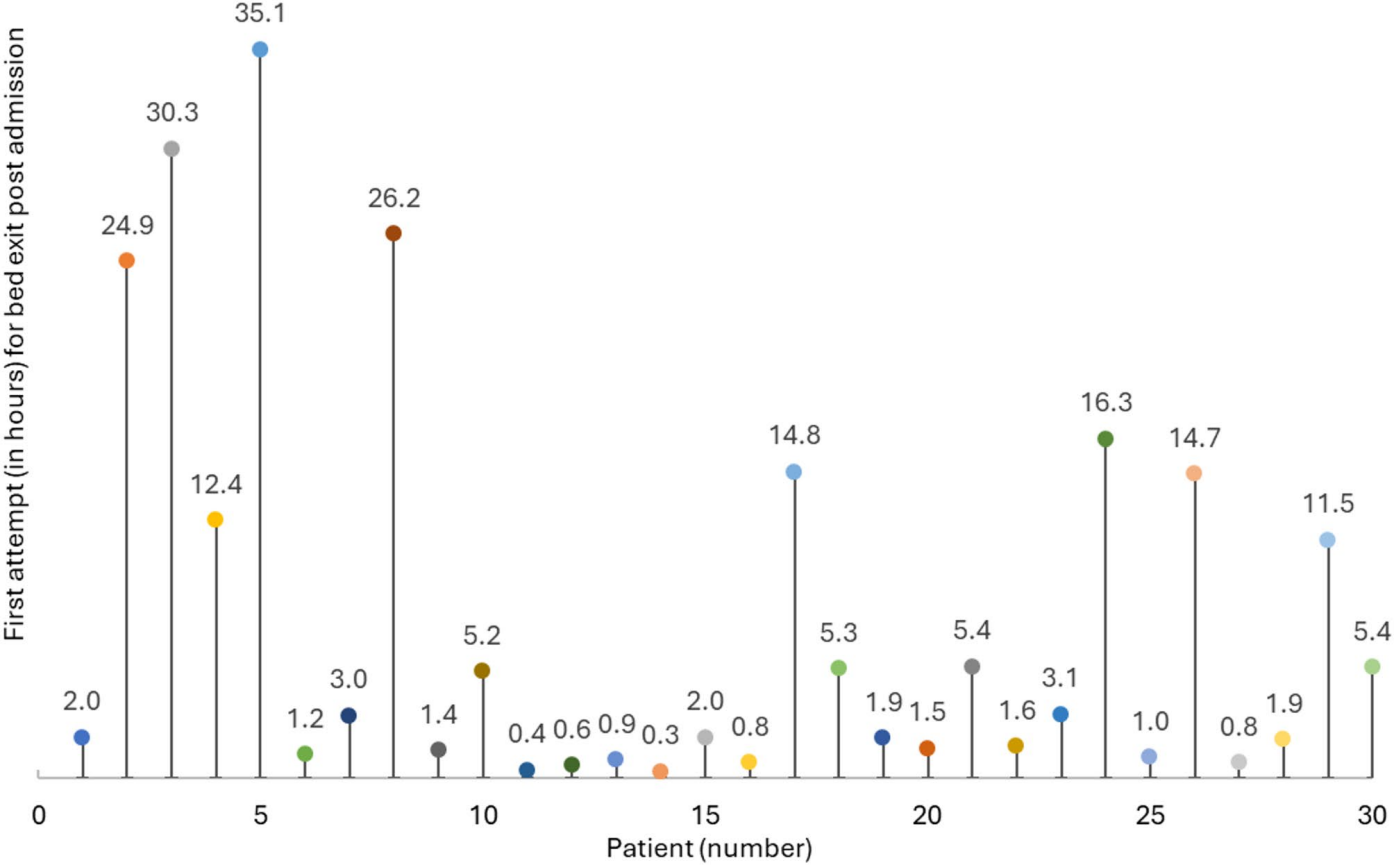
Patients first attempt for bed exit (in hours) post admission (*n* = 30)

Figure 4 illustrates the time (in hours) from admission to the first bed-exit attempt among 30 patients. Each data point represents an individual patient, with the vertical position indicating the elapsed time before the initial bed-exit attempt. The data demonstrate considerable variability: some patients attempted to leave the bed within minutes of admission, while others did not do so for more than a day. The shortest recorded time was 0.3 h, and the longest was approximately 35.1 h. A number of patients-initiated bed exits relatively early (within 1 to 5 h), whereas a few outliers exhibited delayed attempts exceeding 24 h. Patients were monitored for a median duration of 3 days (IQR: 1.0–6.0). The system detected a median of 19 bed-exit events per patient (IQR: 1.0–85.0). The median time to the first bed-exit attempt post-admission was 150 min (IQR: 20.0–2103.0). Across all patients, the median number of bed-exit attempts was 117 (IQR: 20.0–2103.0). The median response time to alerts, defined as the interval between alert activation and staff presence detection was 21 seconds (IQR: 4.0–75.0). These findings highlight the heterogeneity in mobility patterns among patients in the stroke unit, underscoring the importance of individualized monitoring and tailored fall prevention strategies.

The implementation of the SMART AI Patient Sitter system enhanced patient safety outcomes. During the monitoring period, only one fall was recorded under the system’s supervision, compared to six falls reported in the same ward in 2023, reflecting an 83.33% reduction in fall incidents. The recorded fall incidence involved a 53-year-old Malay male patient classified as moderate risk of fall. The incident occurred while the patient was sitting at the edge of the bed and made a sudden movement while attempting to stand, with the presence of his relatives during visiting hours. Clinical staffs in the ward responded promptly, attending to the patient within 29 seconds. Notably, all the study participants were alive at their time of discharge.

Table 2 summarizes the performance metrics of the SMART AI Patient Sitter system in detecting bed-exit attempts. During the study duration, the system detected and triggered a total of 1,439 alerts related to potentially risky events. Investigators responded to 732 of these alerts, representing 50.9% of the total. Notably, despite intervention occurring in only half of the detected events, the system was still associated with a significant decline in fall occurrences. This suggests that early detection and real-time alerting contributed meaningfully to fall prevention efforts.Of the 1,439 alerts generated during the monitoring period, 67 were determined to be false alarms, resulting in a false alert rate of 4.66%. Notably, there were no missed detections, indicating a 100% detection rate for bed-exit events. The system achieved an alert accuracy of 95.34%, demonstrating high reliability in identifying relevant events while minimizing unnecessary notifications, which is an important factor in reducing alert fatigue commonly associated with traditional monitoring systems.

**Table 2:** Performance metrics of the SMART AI patient sitter system for bed-exit attempt detection

Metric	Value	Description
Total bed-exit alerts triggered	1,439	Number of alerts generated by the system indicating potential bed exits
Attended alerts	732	Alerts that were responded to or verified by investigators
Confirmed false alarms	67	Alerts found to be inaccurate upon investigator verification
False alarm rate	4.66%	(67 ÷ 1,439) × 100
Missed detection rate	0%	No actual bed exits were missed by the system

## Discussion

This study investigated the implementation of fall detection using the motion sensor-based SMART AI Patient Sitter system to both detect and prevent falls in a cohort of hospitalized post-stroke patients. Following implementation, the system was associated with 83.33% reduction in patient falls, indicating its potential to enhance safety through accurate and timely monitoring. Although a formal sample size calculation was not conducted prior to the study due to its exploratory design, a post hoc power analysis based on the observed reduction (from six falls to one) yielded a statistical power exceeding 80%. This suggests that the primary outcome is reliable despite the limited sample size.

Globally, studies indicate that stroke patients have a significantly higher risk of falling compared to the general population, with incidence rates ranging from 14 to 65% in hospital settings and up to 73% in community-dwelling stroke survivors within the first year after a stroke [5, 18]. A study conducted in Thailand, reported that 15.9% of stroke patients experienced at least one fall during their rehabilitation period, with an incidence rate of 3.44 falls per 1,000 patient-days. Notably, most falls occurred during the daytime, particularly in bathrooms and by the bedside, often during transfers and walking [19]. A study from Singapore reported that patients with moderate disability may be more prone to falls than those with severe impairment, possibly due to increased mobility without adequate support [20]. This is similar to the fall incidence reported in this study, which involved a 53-year-old male who may have had moderate disability and increased mobility.

The risk of falls in stroke patients is influenced by several factors, including impaired balance, muscle weakness, cognitive deficits, and the use of certain medications. Given the substantial burden of stroke in Malaysia, with patients being approximately 10 years younger than those in developed countries (62.8 vs. 72.3 years), addressing fall prevention is crucial. Implementing targeted rehabilitation programs and fall prevention strategies can help mitigate this risk and improve the quality of life for stroke survivors in Malaysia [21].

The prevalence of patient falls is widely recognized as a key hospital quality indicator, reflecting patient safety, quality of care, and overall hospital performance [22]. Falls, particularly among vulnerable populations like stroke patients, can lead to serious injuries, prolonged hospital stays, increased healthcare costs, and higher morbidity rates [18].

From our study, first attempt to get out of bed post-admission occurred at a median time of 150 min (IQR: 20.0–2103.0). Duration until first-time bed exit in stroke patients is influenced by several factors, including stroke severity, comorbidities, and rehabilitation protocols. Evidence from the AVERT (A Very Early Rehabilitation Trial) study demonstrated that patients in the early mobilization group achieved their first out-of-bed activity at a median of 18.5 h post-stroke, compared to 22.4 h in the standard care group, highlighting the feasibility of initiating mobilization within the first 24 to 48 h following stroke onset, provided the patient is medically stable [23]. However, for patients with moderate to severe strokes, impaired consciousness, or medical instability, the timing of first bed exit may be delayed to between 3 and 7 days or longer [24]. Current guidelines by the American Heart Association/American Stroke Association recommend individualized assessment to determine appropriate mobilization timing, emphasizing a balance between early rehabilitation and patient safety [25]. Overall, early but safe mobilization is a key goal in acute stroke care to promote recovery.

Many healthcare organizations, including the World Health Organization (WHO), The Joint Commission (TJC), and the Agency for Healthcare Research and Quality (AHRQ), include fall rates in their patient safety goals, emphasizing their importance in evaluating hospital care standards. Common hospital falls indicators include the fall rate per 1,000 patient-days, the percentage of patients experiencing falls with injuries, and recurrent falls among high-risk patients, such as those recovering from stroke or surgery. To reduce fall risks, hospitals implement strategies such as regular fall risk assessments using standardized tools like the Morse Fall Scale, the use of assistive devices and bed alarms, proper staff training and patient education, environmental modifications like non-slip floors and grab bars, and medication reviews to minimize side effects that contribute to falls. Effective fall prevention is essential to improving patient outcomes and ensuring high-quality healthcare delivery.

The SMART AI Patient Sitter system is designed to integrate effectively into existing ward operations through the use of overhead-mounted sensors and centralized AI servers, enabling continuous monitoring without disrupting patient comfort or clinical workflows. Despite only 50.87% of alerts receiving direct staff intervention, the reduction in fall incidents indicates that the system provides proactive support in identifying and mitigating potential risks, rather than relying solely on reactive responses.

The system’s ability to detect sudden and unpredictable movements such as those observed in the single fall incident may benefit from further enhancement through integration with clinical data sources. Currently, the SMART AI Patient Sitter system assesses risk primarily based on behavioral cues. Its effectiveness could be improved by incorporating physiological monitoring, such as vital signs and early warning scores. For instance, if a patient displays abnormal physiological indicators (for example: elevated heart rate, blood pressure variability, or changes in respiratory rate), the system could dynamically adjust the patient’s fall risk classification in real time. This would enable the AI to increase alert sensitivity and prioritize monitoring for individuals exhibiting signs of physiological instability, thereby improving the likelihood of preventing falls that may occur too rapidly to be anticipated through behavioral analysis alone. Integrating both behavioral and physiological data would support a more responsive and adaptive system, particularly in high-risk clinical scenarios.

Based on these findings, the SMART AI Patient Sitter demonstrates potential for broader implementation across various care settings, including stroke, dementia, medical, surgical, orthopedic, oncology, and geriatric wards, as well as in home-based eldercare environments. Further large-scale, multi-center studies are recommended to validate these outcomes and inform future implementation and policy decisions.

### Strengths and limitations

This study represents one of the earliest real-world evaluations of an AI-powered fall prevention system implemented in a Malaysian public hospital, providing practical insights into its clinical effectiveness, operational feasibility, and integration within a high-risk acute stroke unit. Unlike simulation-based or retrospective studies, this prospective deployment captured real-time patient outcomes, alert accuracy, and staff response patterns over an extended observation period.

Importantly, the system maintained patient privacy and minimized unnecessary alerts, addressing two common challenges in the adoption of AI technologies in clinical environments. Its non-contact design, compatibility with existing clinical workflows, and function as a proactive early-warning tool further underscore its potential value in enhancing patient safety.

Despite the encouraging findings, this study has several limitations. First, it was conducted in a single hospital unit, which may limit the generalizability of results to other settings with different infrastructure, staffing models, or patient populations. Second, the absence of a formal control group and randomization introduces the possibility of selection bias and unmeasured confounding variables. Additionally, the system’s ability to detect rapid and unpredictable movements such as those seen in the single fall incident remains an area for improvement. Integration with physiological monitoring and automated risk score adjustments may enhance responsiveness and risk stratification in future iterations.

Finally, although a reduction in fall incidents was observed, other potential outcomes such as changes in length of stay, rehabilitation progress, or cost-effectiveness—were not assessed and warrant further investigation.

## Conclusions

Stroke patients face a significant risk of falls, influenced by a combination of physical, cognitive, and environmental factors. Effective fall prevention strategies, particularly implementing a tailored interventions such integrating the SMART AI Patient Sitter system in their care, are crucial in reducing fall incidence and improving patient outcomes. To our knowledge, this is the first clinical evaluation of a commercially available AI-powered fall detection and prevention system deployed in a real-world Malaysian public hospital, specifically within a government acute stroke unit. Unlike most regional studies that remain in pilot or simulated environments, this research quantifies real-time patient monitoring, alert generation, and staff response intervals in an operational setting. The SMART AI Patient Sitter system integrates seamlessly into hospital workflows, enabling informed consent-based activation, uniform-based staff recognition, and real-time system toggling: features rarely documented in clinical AI literature. Notably, it offers privacy-preserving monitoring through blurred, non-identifiable images while delivering actionable alerts via dual alert panels. Furthermore, the study provides novel behavioral insights by analyzing AI-derived event logs on bed-exit patterns and response times, offering a proactive, non-intrusive approach to enhancing patient safety. These contributions address a critical gap in regional clinical research and demonstrate the feasibility of AI-based fall prevention in resource-constrained healthcare systems.

## Data Availability

No datasets were generated or analysed during the current study.

## Abbreviations

AHRQ: Agency for Healthcare Research and Quality
AI: Artificial intelligence
ASUHSJ: Acute Stroke Unit, Hospital Seberang Jaya
AVERT: A Very Early Rehabilitation Trial
CCTV: Surveillance camera
CNN: ResNet-based convolutional neural network
ECG: Electrocardiograph
EMG: Electromyography
IMUs: Inertial measurement units
IQR: Interquartile range
LAR: Legally authorized representative
LSTM: Long short-term memory
MEMS: Micro-electromechanical systems
RNNs: Recurrent neural networks
SOTA: State of the art
TJC: The Joint Commission
WHO: World Health Organization
YOLO: You Only Look Once
